# CED-4 CARD domain residues can modulate non-apoptotic neuronal regeneration functions independently from apoptosis

**DOI:** 10.1038/s41598-019-49633-9

**Published:** 2019-09-16

**Authors:** Guoqiang Wang, Lin Sun, Christopher P. Reina, Isaac Song, Christopher V. Gabel, Monica Driscoll

**Affiliations:** 1 Department of Molecular Biology and Biochemistry, Rutgers University, Piscataway, NJ 08854 USA; 2 Department of Physiology and Biophysics, Boston University School of Medicine, Boston, MA 02118 USA

**Keywords:** Cell signalling, Cell growth, Regeneration and repair in the nervous system

## Abstract

A major challenge in regenerative medicine is the repair of injured neurons. Regeneration of laser-cut *C*. *elegans* neurons requires early action of core apoptosis activator CED-4/Apaf1 and CED-3/caspase. While testing models for CED-4 as a candidate calcium-sensitive activator of repair, we unexpectedly discovered that amino acid substitutions affecting alpha-helix-6 within the CED-4 caspase recruitment domain (CARD) confer a CED-4 gain-of-function (gf) activity that increases axonal regrowth without disrupting CED-4 apoptosis activity. The *in vivo* caspase reporter CA-GFP reveals a rapid localized increase in caspase activity upon axotomy, which is absent in *ced-4* and *ced-3* loss-of-function mutants but present in the *ced-4*(*gf*) mutant. The *ced-3* loss-of-function mutation can significantly suppress the axonal regrowth of the *ced-4*(*gf*) mutant, indicating that CED-4(gf) regeneration depends on CED-3 caspase. Thus, we identified a subdomain within the CED-4 CARD that regulates the dynamic and controlled caspase activity required for efficient regeneration.

## Introduction

Spinal cord injury (SCI) commonly causes permanent loss of sensation and strength below the injury site. Currently, complete neurological recovery after treatment occurs in less than 1% of cases^1^, underscoring the urgent need for understanding the molecular mechanisms that can promote neuronal regeneration. *Caenorhabditis elegans* is a powerful animal model for the study of neuronal regeneration^2–4^, as the transparent body and documented neuronal network map enable axotomy of specific neurons in physiological context. The characterization of genetic requirements for regeneration in this simple model has been extensive. To date, studies in *C*. *elegans* have revealed several conserved molecular pathways that regulate axonal regrowth consequent to injury^2,4^, one of which unexpectedly involves the core apoptosis pathway^5^.

CED-3 (a caspase cysteine-aspartate protease) and CED-4 (apoptosis protease activating factor-1 Apaf-1 homolog) are required for all apoptotic cell deaths in *C*. *elegans*^6^. The *C*. *elegans ced-4* gene can produce two different CED-4 isoforms via alternative splicing, CED-4S and CED-4L^7^. In apoptosis activation, four sets of CED-4S/CED-4L dimers form the apoptosome to recruit CED-3 to a micro-environment that facilitates autocatalytic cleavage and converts CED-3 into a functional death caspase^8–10^.

CED-3 and CED-4 are also required for efficient regeneration of axotomized neurons^5,11^. How the CED-3 and CED-4 activities are distinctly regulated to promote repair rather than death is not understood. We have speculated that calcium fluxes regulate this process because: (1) a neuronal calcium wave is an immediate response to axotomy^5^; (2) ER calcium-store protein calreticulin (CRT-1) is also required for efficient regeneration and may act upstream of CED-3 in the regeneration pathway^5^; and (3) Ca^2+^ released from endoplasmic reticulum (ER) has been shown to promote axonal regrowth^12^. Interestingly, the CED-4 primary sequence was originally noted to feature some primary sequence homology to EF-hand Ca^2+^ binding domains^13^. We hypothesized that axonal injury might promote Ca^2+^ binding to CED-4 to induce localized CED-3 activation, which would transiently and locally promote axonal regrowth^11^. To test this model, we disrupted the CED-4 EF-hand-related sequences using CRISPR and tested axonal regeneration in the CED-4 “EF-hand” mutants. In addition, we developed the use of the fluorescent caspase reporter, CA-GFP, to measure subcellular caspase activity in an axotomized neuron. Contrary to our model, disruption of one “EF-hand” motif actually increased regeneration, via a mechanism that depends upon CED-3 caspase. Our findings define a CED-4 subdomain situated apart from characterized CED-9 or CED-3 binding regions that has a regeneration-specific role in injury repair.

## Results

### Disruption of the two EF hand-like domains of CED-4

**Figure 1:**
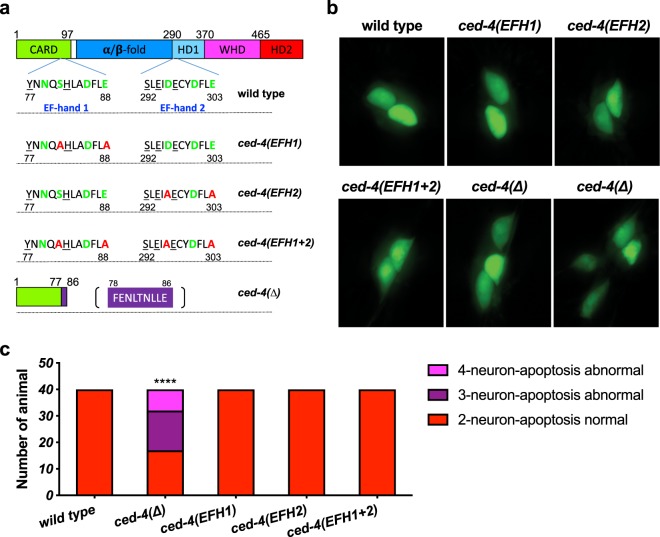
CED-4 EF-hand mutations do not disrupt CED-4 apoptosis functions. (**a**) Amino acid substitutions in the predicted CED-4 Ca^2+^-binding domains, EF-hand 1 (EFH1) and EF-hand 2 (EFH2) were introduced via CRISPR genome editing. Amino acids labeled green are potential Ca^2+^-binding amino acids; in each domain, we replaced two of these residues with alanine (red label). *ced-4*(*∆*) is a likely null allele that could potentially only express a partial CARD domain followed by a random short sequence (labeled purple). Actual allele names are: EFH1 *ced-4*(*bz401*); EFH2 *ced-4*(*bz410*); EFH(1 + 2) *ced-4*(*bz406*); ∆*ced-4*(*bz404*); CARD: caspase recruitment domain*;* HD: Helical domain; WHD: Winged-helix domain. (**b**) Representative pictures of the tail touch receptor neurons labeled by P_*mec-4*_GFP in the background of various *ced-4* alleles. In WT, two PLM tail neurons survive; in *ced-4* null mutants, as many as two additional neurons survive and differentiate, so 3–4 cells may be visualized. (**c**) The PLM tail touch receptor neuron count for *ced-4* mutants, n = 40 animals for each group in one trial. ****p < 0.0001 as compared to the wild type, Chi-square test.

The primary sequence of *C*. *elegans* CED-4 was originally suggested to include two EF-hand-like domains distinguished by an array of oxygen-containing side chain residues similar to a typical EF-hand Ca^2+^-binding domain (Figs 1a and s1a)^13^. EF-hand-like 1 (EFH1) is situated at residues 68–96, positioned in the CED-4 caspase recruitment domain (CARD). EF-hand-like 2 (EFH2) is situated at residues 283–311 of CED-4S (or 305–333 of CED-4L), positioned between the α/β-fold and helix domain 1 (Fig. 1a)^10,13^. Both EF-hand related regions are included in the CED-4S and CED-4L alternative splice isoforms^7^. Although comparing the CED-4 EF hand domain structures to now solved structures of canonical EF hand domains suggests limited similarities to canonical EF hand structures (see details in Fig. S1b), we remained curious about the potential of these sites to link calcium fluxes associated with axotomy to regenerative outgrowth.

With an initial interest in testing the importance of CED-4 EH-hand-like domains in neuronal regeneration, we used CRISPR technology to engineer specific changes in the *ced-4* coding sequence. For EFH1 disruption, we engineered substitutions S81A and E88A, which, for simplicity, we refer to as *ced-4*(EFH1); for EFH2 disruption, we engineered substitutions D296A and E303A, which we refer to as *ced-4*(EFH2). We also generated a strain that featured both substitution clusters, *ced-4*(EFH1 + 2). To compare outcomes with a likely null *ced-4* allele, we also used a *ced-4*(*∆*) strain that harbors a 358 bp deletion beginning within the CARD domain at Y77 that is followed by a random 21 bp insertion before a termination codon is reached.

### CED-4 “EF-hand” amino acid substitutions do not disrupt apoptotic functions

CED-4 plays a core role in activation of the apoptotic pathway in *C*. *elegans* development^14^. In *ced-4* loss-of-function mutants, cells that are normally fated to die, instead live. To address whether the EFH substitutions alter CED-4 apoptosis functions, we tested for cell death defects. In the fully elaborated developmental cell lineages that generate the posterior PLM neurons, the sister cell of the PLM progenitor cell normally undergoes apoptosis^15^. In a *ced-4* cell death mutant, the progenitor sister survives and sometimes divides and partially differentiates, such that 3 or 4 touch neuron-like cells are generated in the mutant sublineage (2 normal and 1–2 undead). The “undead” neurons can be easily visualized as they often express a P_*mec-4*_GFP transgene (in the posterior, expression of this transgene is normally restricted to the two surviving PLM neurons). Thus, the apoptosis defective phenotype is the presence of 3–4 GFP labelled posterior neurons in a P_*mec-4*_GFP-expressing line, rather than two GFP-expressing cells in an apoptosis proficient strain.

We imaged the tail regions of 40 animals from WT, *ced-4*(*∆*), and each *ced-4* EF hand allele to score numbers of fluorescent cells in the tail (Fig. 1b,c). WT consistently showed two PLM tail neurons, and *ced-4*(*∆*) exhibited a high proportion of animals with 3–4 fluorescent cells (~half), consistent with defective apoptosis (Fig. 1c). Interestingly, however, we found that the EFH1, EFH2, and the double EFH1 + 2 *ced-4* mutants had wild type apoptosis profiles, producing no extra surviving neurons (*p* < 0.0001, Chi-square test). We conclude that the amino acid substitutions we tested do not disrupt CED-4 apoptotic functions. Moreover, because the *ced-4*(EFH1), *ced-4*(EFH2), *ced-4*(EFH1 + 2) mutants are WT for cell death, we infer that the alleles encoding the EFH substitution mutants must specify and produce stable CED-4 proteins that can execute apoptosis.

### The CED-4 EFH1 mutant (CED-4 S81A E88A) increases axonal regrowth

**Figure 2:**
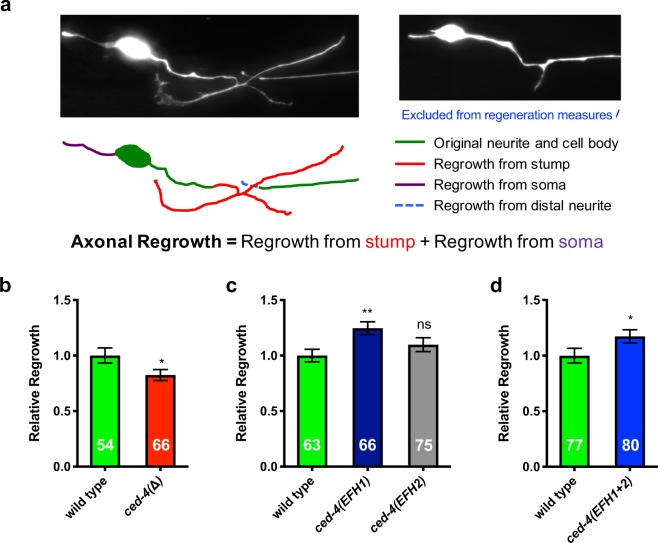
The *ced-4*(*EFH1*) mutant exhibits increased axonal regrowth consequent to axotomy. (**a**) Depiction of measures of axonal regrowth from ALM touch receptor neurons. (**b**–**d**) The axonal regrowth measures for the *ced-4* null mutant and the engineered EF-hand mutants. Total n numbers are given inside bars. All data come from >3 independent trials, *p ≤ 0.05, **p < 0.01, or ns (not significant) as compared to the *ced-4*(+) wild type, unpaired two-tail *t*-test (**b**,**d**) or one-way ANOVA with Tukey adjustment for multiple comparison (**c**); for (**d**), *p* = 0.0545.

We then examined the impact of the CED-4 EFH substitutions on axonal regrowth consequent to laser axotomy, using the P_*mec-4*_GFP transgene to visualize the touch neurons and their processes. We made a laser cut in the ALM axon ~20 µm from the soma, according to our standard protocol^5,12,16^. The axotomized neuron grew new neurites from both the axonal stump and the soma (Fig. 2a). 24 hours post-injury, we measured and added up axonal regrowth from the stump and from the soma to evaluate the extent of neuronal regeneration. Because of the inability to distinguish newly regenerated tracks that run together with the surviving severed fragment, we did not score the cases in which new neurites appeared to reconnect back to the distal neurite.

We first confirmed that CED-4 is required for efficient axonal regrowth by comparing the *ced-4*(∆) strain to the WT strain (*p* < 0.05, Fig. 2b). We scored defects in regeneration in the *ced-4*(*∆*) mutant, consistent with our previous observations on different *ced-4* alleles^5^. Note that parallel pathways promote regeneration^2,4,17^, and the *ced-4 ced-3* pathway is just one of these^5^, so typically regeneration outgrowth scores are significantly diminished by mutation in pathway genes, but regeneration is not eliminated.

We then examined the axonal regrowth of the EF-hand mutants. We found that the *ced-4*(*EFH2*) mutant exhibited axonal regrowth efficiency similar to WT. Thus, the integrity of the candidate EFH2 site is required for neither apoptosis nor regeneration function. Our analysis of the *ced-4*(*EFH1*) mutant, however, yielded a surprise: *ced-4*(*EFH1*) exhibited an ~25% increase in axonal regrowth (Figs 2c and s2; *p* < 0.0001 for 6 combined trials, a total of 116 neurons from WT and 124 neurons from the EFH1 mutant). The efficiency of axonal regrowth also was increased in the *ced-4*(*EFH1&2*) double substitution mutant (*p* = 0.0545), independently supporting the effect of the EFH1 S81A E88A substitutions (Fig. 2d). To test whether the enhanced regeneration phenotype could be observed in additional neurons, we examined the axonal regrowth of posterior PLM touch neurons consequent to axotomy (Fig. s3). We find that *ced-4*(*EFH1*) also promotes enhanced regenerative outgrowth in injured PLM neurons (*p* < 0.01).

In *C*. *elegans* genetics, loss-of-function null mutations often have the “opposite” phenotype of gain-of-function alleles (for example, *ced-9* loss-of-function alleles promote apoptosis but *ced-9* gain-of-function alleles inhibit apoptosis^18^). Since the *ced-4* null loss-of-function phenotype is less regenerative outgrowth (Fig. 2b and ref.^5^) but the *ced-4*(*EFH1*) phenotype is increased regenerative outgrowth, the EFH1 S81A E88A substitutions can be proposed to reveal a potential novel gain-of-function activity of CED-4 in regeneration.

Note that we do not find evidence of enhanced developmental apoptosis as evaluated by compromised embryonic viability and apoptotic corpse counts in *ced-4*(*EFH1*) (Fig. s4), reinforcing the conclusion that CED-4 regeneration and apoptosis activities can be separated.

### An active site *ced-3* mutation suppresses the elevated axonal regrowth of the CED-4(EFH1) mutant

**Figure 3:**
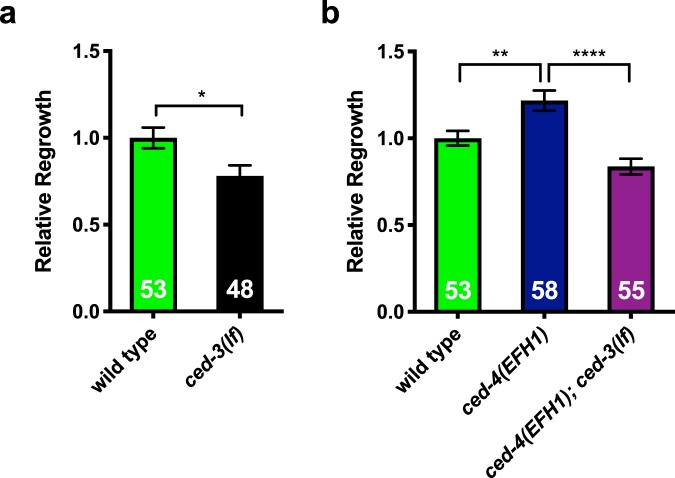
A *ced-3* mutant allele that disrupts the caspase active site suppresses the enhanced axonal regrowth of the *ced-4*(*EFH1*) mutant. (**a**) *ced-3*(*n2433*), lacking the caspase active site (indicated *ced-3*(*lf*)), exhibits reduced regeneration. Data come from >3 independent trials, *p < 0.05, unpaired *t*-test. (**b**) impact of *ced-4*(*EFH1*) on regeneration outcomes. Data come from >3 independent trials, **p < 0.01 or ****p < 0.0001, one-way ANOVA with Tukey adjustment for multiple comparison; numbers of animals scored indicated in bars. Separate graphs are given for time-separated trials.

In the canonical pathway for CED-3 caspase activation, four pairs of CED-4 dimers form the apoptosome^10^. We previously showed, and confirm here, that apoptosis executor caspase CED-3 is required for efficient axonal regrowth consequent to laser axotomy (Fig. 3a). CED-3 likely acts downstream of CED-4 in the regeneration pathway as modest overexpression of CED-3 in the *ced-4 ced-3* double mutant partially rescues the regeneration compromised phenotype^5^. We therefore wondered whether the unexpected increase in regeneration in the *ced-4*(*EFH1*) mutant depends upon *ced-3* caspase. To test if CED-3 is required for the *ced-4*(*EFH1*) gain-of-function increased regeneration phenotype, we constructed a double mutant strain of caspase active site mutation allele *ced-3*(*n2433*) and *ced-4*(*EFH1*). We compared regeneration in WT, *ced-4*(*EFH1*), and the *ced-4*(*EFH1*); *ced-3*(*n2433*) double mutant. We found that the enhanced axonal regrowth in the *ced-4*(*EFH1*) mutant is suppressed by the presence of the *ced-3* caspase active site mutation (Fig. 3b, *p* < 0.0001). We conclude that *ced-4*(*EFH1*) increases regeneration through a CED-3 caspase-dependent mechanism.

### Axotomy-induced *in vivo* caspase activity correlates with axonal regrowth

**Figure 4:**
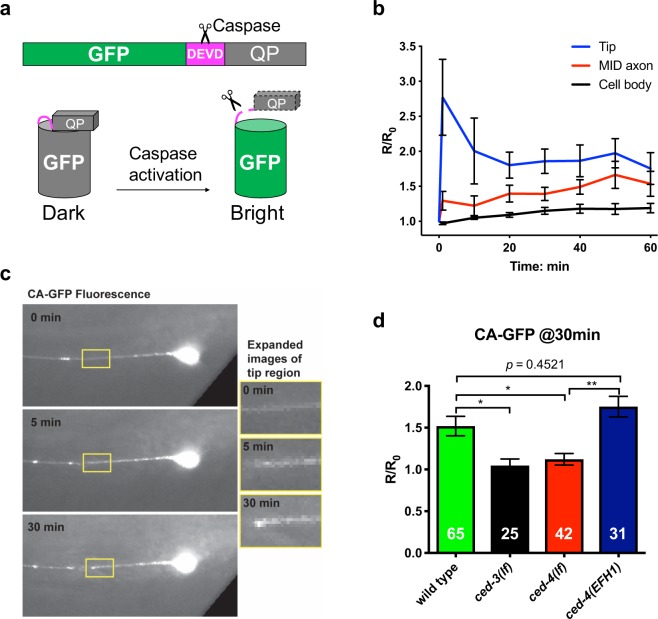
*In vivo* caspase activity assay supports localized CED-3 activation consequent to axotomy, with CED-4(EFH1) capable of caspase activation. (**a**) Cartoon depiction of the CA-GFP reagent for *in vivo* measure of caspase activity. QP: quenching peptide, DEVD is the added consensus caspase cleavage site (purple). When the quenching peptide is removed, GFP fluorescence increases. (**b**) Average time-lapse of *in-situ* CA-GFP measurements for each region indicated. In our studies, we also included a co-expressed mCherry marker in the touch neurons to enable us to normalize GFP values at specific times after axotomy. Measurements are displayed as normalized fluorescence relative to the initial value. R stands for the ratio of CA-GFP/mCherry, and R_o_ is the R before laser severing, error bars represent standard error. (**c**) Images of *in-situ* CA-GFP measurements before laser axotomy at 0 min and after axotomy at 5 min and 30 min. The regions of axon near the point of axotomy (yellow boxes) are expanded for visual comparison (right). GFP signals are weak but not absent at baseline, especially in the soma. Image contrast has been enhanced to aid visualization. For accurate quantification, CA-GFP intensity measurements were normalized using simultaneous images of co-expressed mCherry (see Methods). (**d**) CA-GFP measurements of the severed axon near the point of axotomy, 30 min post axotomy. Measurements are displayed as normalized fluorescence relative to the initial value (R_o_, before laser severing). *p < 0.05 or ns (not significant), one-way ANOVA with Tukey adjustment for multiple comparisons.

The fact that *ced-4*(*EFH1*) enhances regeneration via the CED-3 caspase suggests that caspase activity should increase in the *ced-4*(*EFH1*) background, at least in response to axotomy. Measuring caspase activity *in vivo* is challenging, although reagents such as dark-light fluorescent reporter CA-GFP (Fig. 4a) have been used for such a purpose in mammalian cells^19^. Caspase-dependent cleavage of a DEVD target sequence within CA-GFP releases a fluorescence-quenching peptide in the CA-GFP construct; caspase activity thus leads to fluorescence by eliminating an inhibitor^19^. We co-expressed CA-GFP (dark in the absence of caspase activity) and red fluorescent protein mCherry in the touch receptor neurons (mCherry enables visualization of the axon for laser surgery and normalization of signals). We performed laser surgery 60 μm from the ALM cell body and acquired images in the green and red fluorescence channels immediately before laser surgery and every ten minutes thereafter (Fig. 4b,c).

We find that consequent to axotomy, CA-GFP fluorescence rapidly increases within close proximity of the severed end, while a slower, weaker increase can be measured at the axon mid-point in the time-lapse measurements for 1 hour following laser axotomy (Fig. 4b,c). We measured only a minimal response in the cell body, consistent with the idea that caspase CED-3 must act locally in the repair process. Thus, although CA-GFP signals were generally low, we nonetheless reliably detected a rapid localized caspase activation in the severed end of the damaged axon. Our observations extend CA-GFP applications to the *C*. *elegans* model.

To verify the dependence of the measured caspase activity on *ced-4* and *ced-3*, we repeated the CA-GFP imaging experiments in mutant backgrounds. In these studies, we measured the CA-GFP fluorescence before laser surgery and at 30 min post-surgery near the point of axotomy. The normalized ratio of CA-GFP fluorescence (30 min/0 min) is shown in Fig. 4d. While the wild-type background shows a ~60% increase at the severed end after 30 min, the increase response was lacking in both *ced-3* and *ced-4* mutants (Fig. 4d). This result is consistent with localized CED-3 caspase activation via a CED-4-dependent mechanism consequent to axotomy^5^.

We next evaluated CA-GFP-reported caspase activation when CED-4 included EFH1 S81A E88A substitutions. Consequent to laser axotomy, *ced-4*(*EFH1*) exhibited caspase activity similar to WT, with an ~70% increase in CA-GFP fluorescence (Fig. 4d). Thus, analysis of CA-GFP signals supports caspase activation in the *ced-4*(*EFH1*) background, consistent with genetic dependence of *ced-4*(*EFH1*) on *ced-3* activity for regeneration enhancement.

## Discussion

**Figure 5:**
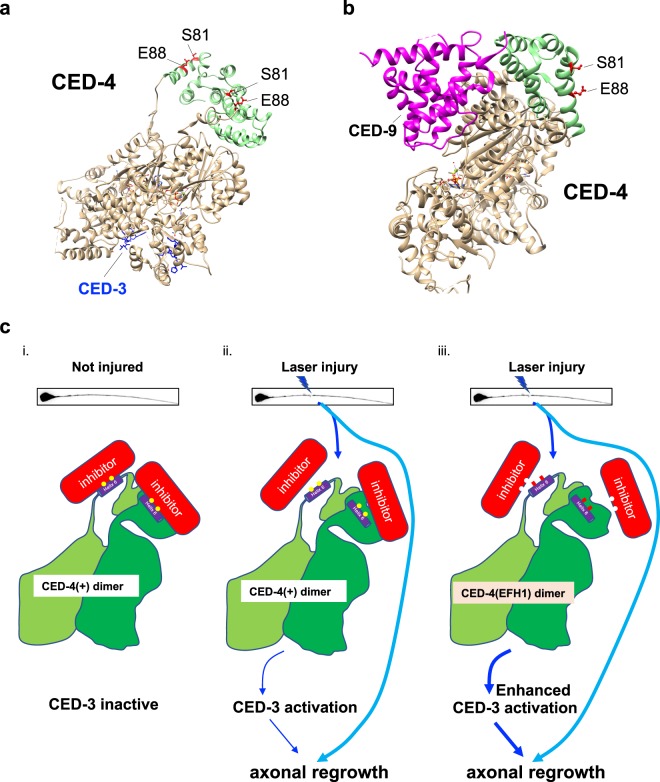
CED-4 S81 and E88 face away from the CED-3 or CED-9 interaction sites that regulate apoptosis, but might be available to interact with a factor that licenses transient CED-3 activation for localized repair. (**a**) Ribbon structure shows a CED-4 dimer (most residues in tan, but CARD domain in green with S81 and E88 highlighted in red), with two interacting CED-3 fragment residues (indicated in blue) as determined from a crystal structure. Note that the CARD domain is positioned apart from the CED-3 interaction domain and that S81 and E88 are oriented away from the main structure such that the subdomain that includes S81 and E88 might interact with another protein (or another region of a dynamic CED-4). (**b**) Ribbon structure shows a partial CED-4 dimer with one CARD domain depicted (CARD domain green, with S81 and E88 in red), binding to the CED-9 protein (magenta). Note that the CARD domain helix 6 containing S81 and E88 is positioned apart from the CED-9 interaction domain, with those AA positions facing outward from the protein. (**c**) A proposed model of CED-4 non-apoptotic function in neuronal repair shows (i) the two essential amino acids (S81 and E88 shown as yellow dots) in CED-4 helix 6 binding to a hypothetical inhibitor (red, indicated as a protein but could be an ion-binding site associated with conformational change); two green shades of CED-4 represent S and L isoforms. Upon injury (ii), the inhibition might be transiently relieved, enabling CED-4 to locally activate CED-3 caspase. Note that known apoptosis inhibitor CED-9 binds to the opposite side of the CARD helix 6 domain and is unlikely to be directly involved at the helix 6 sites. (iii) The substitution of alanine for S81 and E88 may reduce the affinity of the inhibitor to bind to the CED-4 dimer and subsequently increase the capacity to activate CED-3 in response to injury stress. When S81 and E88 in the CARD domain are absent, the interaction with the inhibitor is weakened and regeneration outgrowth is increased in a CED-3 caspase-dependent mechanism under injury conditions.

Precise regulation of protease function is critical for cellular homeostasis, yet little is understood about the subtleties of limited protease activity *in vivo*. Particularly mysterious is the question of how apoptosis executor caspases are repurposed and precisely regulated to execute beneficial non-apoptotic outcomes in the cell. In probing the mechanism of non-apoptotic functions of CED-4/Apaf-1 and CED-3/caspase in *C*. *elegans* neuronal regeneration, we unexpectedly identified substitutions in CED-4 that can uncouple regeneration activity from apoptosis function. In the context of published structures of CED-4, CED-4-CED-3/caspase complexes, or CED-4 CED-9/BCl-2 complexes, our data highlight a CED-4 CARD subdomain through which CED-4 might modulate localized and limited caspase activation in non-apoptotic functions (model in Fig. 5c).

### CED-4 domains in apoptosis vs. regeneration

Most existing *C*. *elegans* apoptosis mutations were isolated by virtue of their cell death phenotypes^18,20–23^ and therefore must disrupt apoptosis by definition. The CRISPR-generated *ced-4* S81A E88A allele, although altered for regeneration, can execute apoptosis functions (Fig. 1b,c) and does not appear to have gain-of-function activities in apoptosis (Fig. s4). To the best of our knowledge, this is the first identification of a *ced-4* allele that separates non-apoptotic function from apoptotic function and exerts gain-of-activity for the non-apoptotic function.

Interestingly, the apoptosis-independent role of CED-4 in regeneration is one of several examples of non-apoptotic CED-4 function in *C*. *elegans*, including CED-4 action in a *ced-3*-requiring ROS-responsive pathway that mediates longevity of some mitochondrial mutants^24^, and *ced-3-*independent hypoxic preconditioning^25^ and DNA-damage-induced cell cycle arrest^26^. Fly CED-4/Apaf-1 homolog DARK functions in the caspase-requiring process of spermatid individualization^27^. Moreover, mouse Apaf-1 mutants have defects in olfactory neuron development and axon outgrowth, but not in neuron numbers^28^, suggesting potential for a conserved application of CED-4/Apaf- activity in neuronal outgrowth apart from cell death. Future experimental focus on the Ser81 Glu88 associated domain we identify here should extend details of the molecular mechanisms by which CED-4/Apaf-1 family members engage programs of cell health and maintenance.

How might differential regulation (apoptosis vs. regeneration) of CED-3 caspase by CED-4 be accomplished? Elegant structural studies on CED-4, and on CED-4/CED-3 or CED-4/CED-9 complexes, have been published^10,29–32^, providing models with which we can examine the potential impact of the Ala substitutions for Ser81 and Glu88 in CED-4 gain-of-function activity. We first considered impact of Ser81Ala and Glu88Ala in the solved crystal structure of the CED-4 CARD domain. As visualized in a ribbon diagram, Ser81 and Glu88 align on one helical surface (helix 6) that is hydrophilic in character and appears to face outward from the CARD domain where it could be easily accessible to other proteins or ions (Fig. 5). Examination of structural data for CED-4/CED-3 or CED-4/CED-9 complexes (Fig. 5a,b) suggests why Ser81 and Glu88 do not interfere with apoptosis—the Ser81 and Glu88 are situated in a domain that does not interface with other CARD domain regions, with CED-3 caspase, or with CED-9/Bcl-2. The fact that the line of residues in helix 6 does not appear to make contacts critical for apoptosome formation is consistent with the fact that the S81A G88A substitutions do not change CED-4 capacity to promote apoptosis.

Of note, the relevant alpha-helical domain appears to be exposed to the outside of the CARD domain, where its charged residues might interface with an interacting protein (model in Fig. 5c). Sequence alignment of the CED-4 orthologs in *Caenorhabditis* species shows that the specific identities of the S81 and E88-equivalent amino acids vary but are hydrophilic in most cases (Fig. s5c). Other CED-4/Apaf-1 family members, such as DARK from fly and Apaf-1 from human, feature similar hydrophilic helices (Fig. s5a,b), suggesting this conserved structural feature may be used for caspase modulation across phyla.

### CA-GFP can report on caspase activation in *C*. *elegans*

We report on the first application of dark-to-bright caspase-activatable GFP in *C*. *elegans*. The CA-GFP reagent harbors a hydrophobic quenching peptide that prevents proper folding of the GFP fluorophore; the inhibitory peptide is linked to the GFP domain via a caspase consensus target site^19,33^. When caspase is active, the inhibitory domain is cleaved from the protein, enabling the GFP to fold and fluoresce for signal detection. Our data show CA-GFP can report localized caspase activation in *C*. *elegans* neurons. CA-GFP imaging enabled measurement of localized caspase activity in the damaged neurite, verified signal dependence on *ced-4* and *ced-3*, and revealed recovery of caspase activity in the *ced-4*(*EFH1*) mutant. Although the noise level was high in our study, our focus was on tiny (~200 nm) diameter processes^4^, and thus more robust CA-GFP signals might be expected for developmental apoptosis. CA-GFP might be exploited to facilitate future study of apoptotic death regulation and general caspase regulation. Moreover, because fluorescent signal generation is specifically dependent upon the cleavage of consensus sites within the reporter linker, the CA-GFP reagent might readily be modified to contain consensus target sites for the study of regulation of other proteases in *C*. *elegans*.

### Reconsidering a model in which calcium binds to EFH-like domains to activate CED-4 in regeneration

Given the documented role of calcium in the neuronal response to axotomy^34^, the role of calcium-storing calreticulin in the CED-4/CED-3 regeneration pathway^5^, and the initial suggestion that the CED-4 primary sequence might include EF-hand like calcium binding sites^13^, we sought to evaluate the functional requirements for the CED-4 EF-hand-like sites in the activation of regeneration. We found that changes in neither of the two CED-4 EFH-like sequences blocked regeneration (distinct from the *ced-4* null phenotype), which, on the simplest level, does not support a model for a direct Ca^2+^-binding role for these domains during transient activation of CED-4. Moreover, in the years since the original ground-breaking cloning of *ced-4*^21^, detailed structural data on both CED-4 and numerous EF hand domains became available^10,29–32^. Solved structures show that CED-4 does not feature canonical EFH Ca^2+^-binding sites, with EFH1 only distantly related to such motifs and EFH2 not related (Fig. s1).

Although EFH1 appears unlikely to directly bind calcium via a canonical EF-hand domain mechanism, it remains possible that residues in this region are nonetheless required to couple calcium signals to CED-4 activity. How might the CED-4 alpha helix 6 contribute to localized caspase activation consequent to injury? In theory, the gain-of-function phenotype of enhanced regrowth could be the result of disrupting a CED-4 inhibitory domain that modulates non-apoptotic functions, or enhanced regrowth could be the result of a mutation that renders CED-4 constitutively active/less dependent on Ca^2+^ or another regeneration activation signal (model in Fig. 5c). The alpha helix 6 domain might serve to bind a negative regulator that is inactivated by calcium or, alternatively a calcium-sensitive activator might interact via this site. At the same time, an activating factor other than Ca^+2^ might be involved. The identity of the hypothesized binding protein, and the nature of the signal that promotes its binding, remains for future investigation.

In sum, our molecular genetic studies of the gain-of-function *ced-4* S81A E88A mutant suggest that an exposed hydrophilic CED-4 CARD domain helix in which S81 and E88 are situated may constitute a site via which CED-4 modulates non-apoptotic functions, driving limited caspase activation toward constructive cellular ends.

## Methods

### Strain construction and maintenance

The *Caenorhabditis elegans* control P_*mec-4*_GFP strain, ZB4510, was generated after more than five times outcrossing of SK4005 to our N2 wild type strain. We did all genomic editing of *ced-4* genes on the ZB4510 background via CRISPR. We used a ribonucleoprotein protocol for CRISPR^35^. The sequence of crRNA (combined with tracrRNA to guide Cas9 in cutting of the targeted sequence) and ssDNA oligonucleotides (for repair) used in this study are all listed in Supplementary Table 1. We selected F1 roller animals for genotyping of targeted mutations and selected the non-roller F2 to identify the homozygous mutant via PCR-based genotyping. We then confirmed the mutations with sequencing. All mutants were backcrossed at least twice to the reference strain, ZB4510. The detailed information for all strains is listed in Supplementary Table 2.

The CA-GFP coding sequence was transferred from mammalian expression vector pmKate2C into the pPD49.26 vector with a *mec-4* promoter. The P_*mec-4*_CA-GFP was co-injected with P_*mec-4*_mCherry plasmid and gamma irradiated for integration. After backcrossing to our N2 strain several times, the final *bzIs178* [P_*mec-4*_CA-GFP + P_*mec-4*_mCherry] homozygous strain was designated as ZB4019.

All the strains grew under continuous growth on OP50-1 bacteria-seeded nematode growth media (NMG) agar plates at 20 °C for at least 10 generations before being used in any experiments.

*C*. *elegans* is the only animal used in this study. There are no live vertebrate animal or human participants in this study.

### 3D structure presentations

The EF-hand domain 3D structure of human calcineurin is from NCBI’s Molecular Modeling Database (MMDB) with PDB ID: 2P6B. The EF-hand like domain 3D structure of *C*. *elegans* CED-4 came from the NCBI’s MMDB with PDB ID: 4M9X. Other 3D structures used in this study include CED-4 CED-9 complex (PDB: 2A5Y), Apaf-1 (PDB: 5JUY) and DARK (PDB: 1VT4, 3IZ8).

### Tail touch neuron count

We imaged the tail region of animals at the L2 to L4 developmental stage in Z stacks, then we counted the number of tail touch neurons in the Z projected images.

### Laser axotomy and regeneration measurements

We used egg lays to synchronize the life stages of animals for study, picking the early young adults for laser axotomy. The animals were immobilized with beads (diameter: 0.05 Micron, catalog#: 08691, company: Polysciences, Inc.) and a 7–8% agarose pad (catalog#: CA3510-6, company: Denville Scientific, Inc.). The laser axotomy of ALM neurons was performed with an Andor Micropoint Laser on a Zeiss inverted microscope (AX10). The animals were then relocated back to the bacterial seeded NGM agar plate. After ~24 hours culture under 20 °C, the animals were paralyzed with sodium azide and the images of axotomized ALM neurons were acquired in Z stacks. About 40 animals were axotomized for each genotype in every trial. Animals with neurites connected to the distal axon were excluded from the regeneration measurements because we could not visually distinguish new growth from the original neuron in those cases. We added the length of new neurites grown from both cell body and the axotomized stump to evaluate axonal regrowth.

### Laser axotomy and CA-GFP quantification

We performed caspase imaging experiments with animals expressing both CA-GFP (green) and mCherry (red) in the mechanosensory neurons. Both green and red channels were imaged before and after laser surgery of the ALM neuron with 1 sec exposures at the described time points. A Ti:Sapphire infrared laser system (Mantis PulseSwitch Laser, Coherent Inc) (as in ref.^5^) was used for these surgeries. Fluorescence intensities were measured within the regions of interest in both channels. CA-GFP intensity was given as the normalized ratiometric (green–green background/red–red background) change in fluorescence before and after axotomy. Some animals were excluded from the experiments because the background green signal did not allow accurate identification and measurement of the CA-GPF within the axon. CA-GPF activity was measured within 5 μm of the served axon end, a 5 μm segment at the mid axon, and across the entire cell body as indicated in the text.

### Statistics

We used the unpaired two-tail *t*-test was any experiments with only two sample groups. One-way ANOVA was used for any experiments having three or more sample groups with a Tukey adjustment for multiple comparisons. The confidence interval is 95% for all statistical assays.

## Supplementary information

Supplementary information
