# Supplementary material for: CED-4 CARD domain residues can modulate non-apoptotic neuronal regeneration functions independently from apoptosis
Source: Sci Rep. 2019 Sep 16;9:13315. doi: 10.1038/s41598-019-49633-9 (PMC6746752; doi:10.1038/s41598-019-49633-9)
Supplement: Supplementary file 1 — Supplementary information [file 41598_2019_49633_MOESM1_ESM.pdf]

CED-4 CARD domain residues can modulate non-apoptotic neuronal regeneration functions independently from apoptosis

Guoqiang Wang[1], Lin Sun[2], Christopher P. Reina[1], Isaac Song[1], Christopher V. Gabel[2], Monica Driscoll[1,*]
[1]Department of Molecular Biology and Biochemistry, Rutgers University, Piscataway, NJ 08854, USA.
[2]Department of Physiology and Biophysics, Boston University School of Medicine, Boston, MA 02118, USA.
*Corresponding author: Driscoll@Biology.rutgers.edu

Supplementary Methods

Brood size and dead embryo assay. We picked 8 L4 animals into individual NGM agar plates for each trial, and transferred each animal into a new plate in every day. We counted the progeny number and dead embryos from each plate 24 hours after taking away the parent animal. We excluded the sample when the parent animal died or was lost during the gravid period.

L1 apoptotic corps count. We collected gravid adult animals and bleached these animals to release embryos. We then placed the embryos on top of the NGM agar medium. Most of the embryos hatched after about 10 hours under 20℃. We immediately mounted these newly hatched L1 animals onto 7-8% agarose pads and paralyzed with 40mM sodium azide. We acquired the DIC (Differential Interference Contrast) image in z stack with an upright microscope attached with a HAMAMATSU digital camera C11440. To count the number of apoptotic corpses in each animal, we went through the z stack of each animal and only counted the distinctive corpses in the pharynx area.

Supplementary Figures and Figure Legends

Supplementary Figure 1

a

	10		12		14				18			21
	X		Y		Z				-X			-Z
Ca^{2+}-binding loop consensus sequence	**D**		**N**		**S**				**T**			**E**
			**D**		**N**				**S**			
					**D**				**E**			
									**Q**			
									**D**			
									**N**			
EF-hand consensus	O	*	O	*	O	G	*	*	O	*	*	E
CED-4 sequence 1	Y	N	**N**	Q	**S**	H	L	A	**D**	F	L	**E**
	77											88
CED-4 sequence 2	S	L	E	I	**D**	E	C	Y	**D**	F	L	**E**
	292											303

Conserved amino acids are labeled with **bold** letters. Letter "O" stands for the amino acid with an oxygen-containing side chain. *, non-conserved amino acid. Underlined letter stands for deviated CED-4 amino acid from the EF-hand consensus sequence.

b

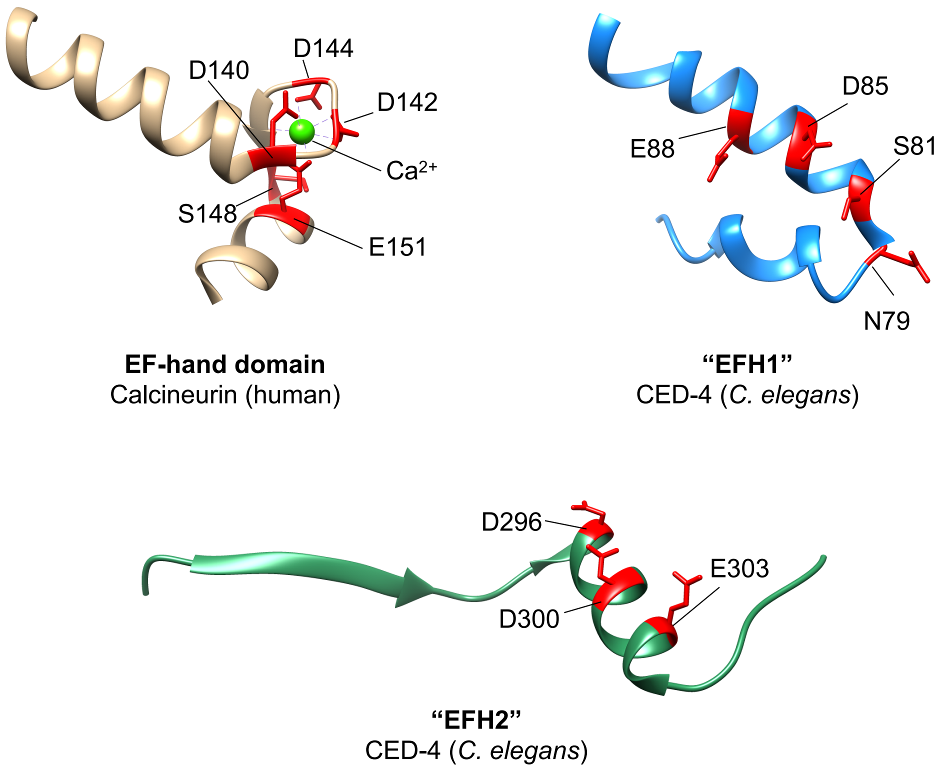

Figure s1. Details on CED-4 EF-hand-like sequences.

a. Original description of the CED-4 relationships to EF-hand domains [1].

b. 3D modeling of the calcineurin EF-hand Ca^{2+}-binding domain structure and the predicted CED-4 EF-hand-like domains folded according to this model. Ribbon diagrams were plotted with the UCSF Chimera software.

The core amino acids of a canonical EF-hand Ca^{2+}-binding domain from human calcineurin [2] are mostly located on the loop of the EF-hand domain and have their side chains directed to the Ca^{2+} (left panel). The CED-4 EFH1 domain can be overlaid on the canonical EF hand structure (Helix-Loop-Helix motif) [3], although the CED-4 core amino acids are not located on the loop, the loop is short, and side chains of the amino acids of interest are not uniformly oriented. EFH2 is less similar to an EF-hand, as EFH2 does not have a helix-loop-helix motif and all the core amino acids are located on the helix [3]. Overall, neither EFH CED-4 domain constitutes a strong match to canonical EF-hand proteins, but given the helix loop similarity, EFH1 could correspond to a non-canonical domain [4].

Supplementary Figure 2

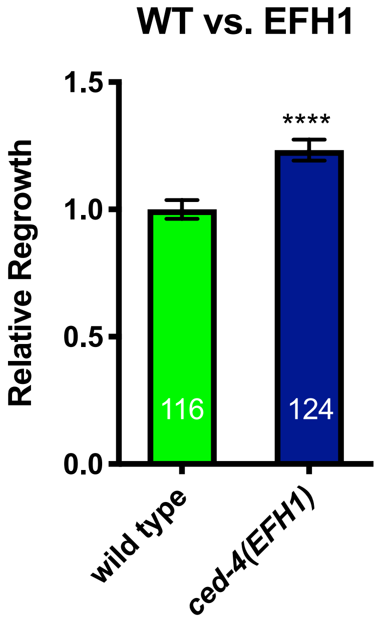

Figure s2. Combined 6 trials of CED-4(EFH1) S81A E88A mutant in the axonal regrowth assay.

Three trials come from Fig. 2c and the other three come from Fig. 3b. **** $p < 0.0001$, unpaired two-tail *t*-test.

Supplementary Figure 3

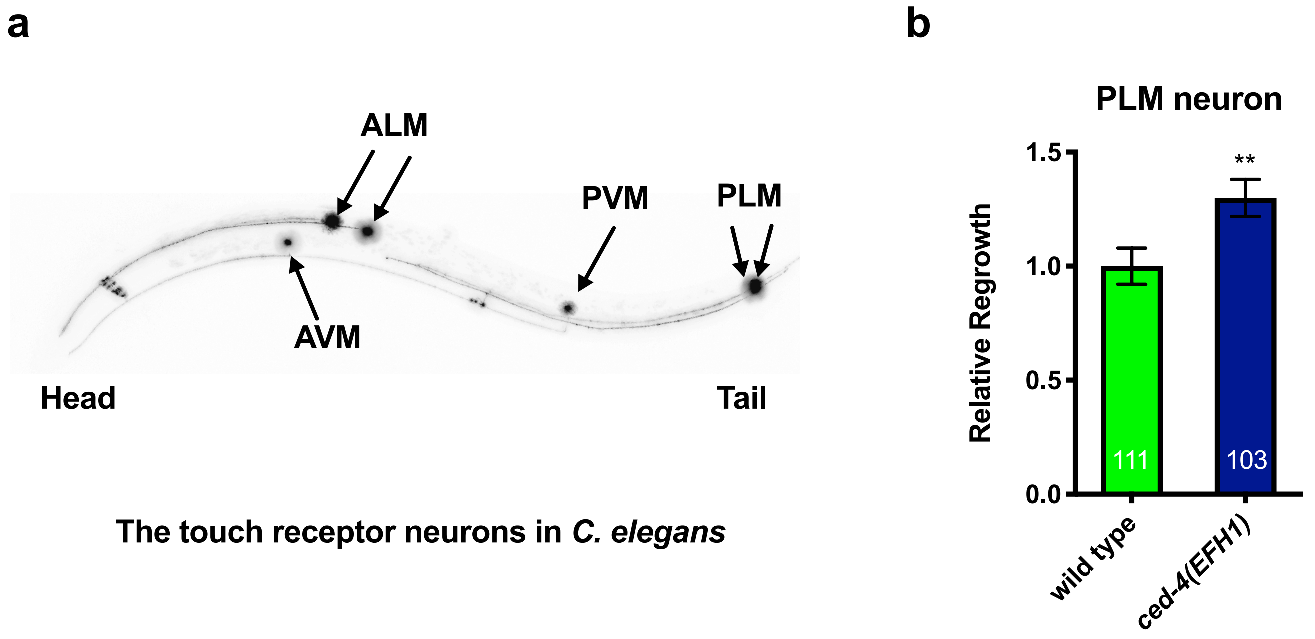

Figure s3. CED-4 EFH1 mutant also promotes axonal regrowth in the PLM neurons.

a. Graph shows the location of six touch receptor neurons in *C. elegans*. ALM: Anterior Lateral Microtubule neurons; AVM: Anterior Ventral Microtubule neuron; PVM: Posterior Lateral Microtubule neuron; PLM: Posterior Ventral Microtubule neurons.

b. Graph shows the axonal regrowth measures of PLM neurons for the *ced-4* EFH1 mutant. Total n numbers are given inside bars. All data come from 5 independent trials, ** $p < 0.01$, as compared to the *ced-4* wild type, unpaired two-tail *t*-test.

Supplementary Figure 4

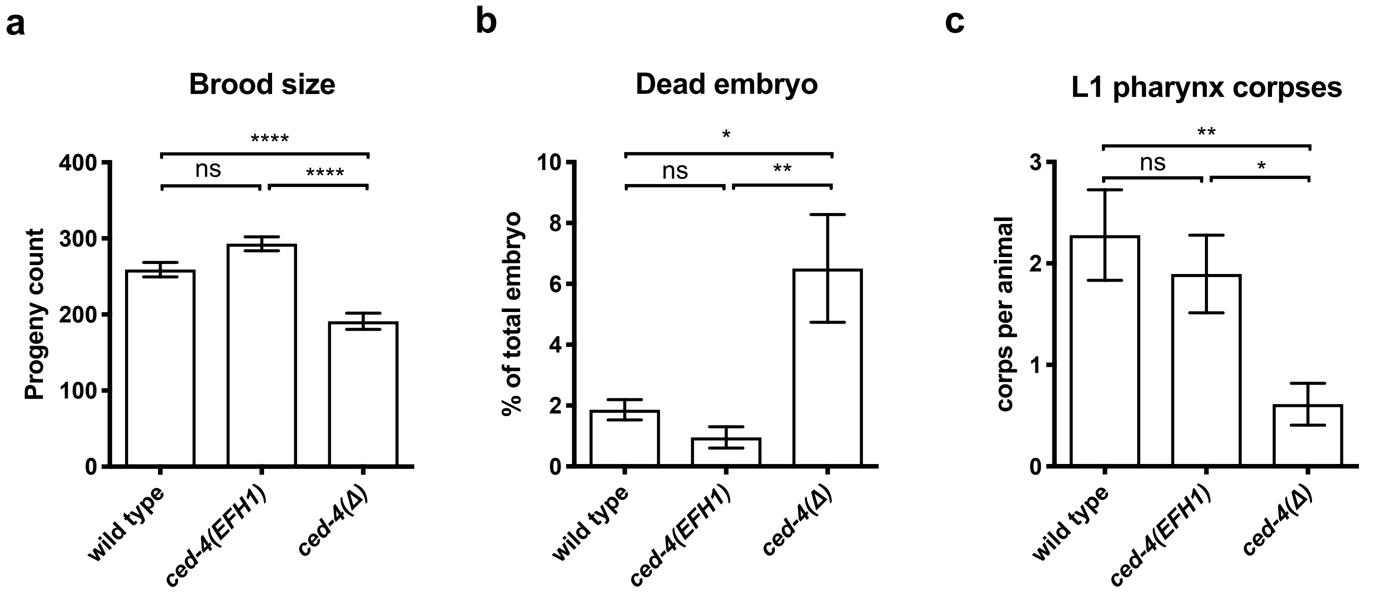

Figure s4. The *ced-4(EFH1)* allele does not induce increased developmental apoptosis.

Our data show that EHF1 mutations are not *defective* for apoptosis (Figure 1) but it remained possible that EFH1 substitutions, which act like gain-of-function for regeneration, might also act as gain-of-function for apoptosis induction, which would increase the numbers of developmental apoptotic deaths. The *ced-9(rf)* phenotype has been well characterized to induce excessive apoptosis, which markedly lowers brood size and embryo viability; elevated numbers of apoptotic cells can be observed in the pharynx area of L1 larvae [5]. Therefore, we scored these three phenotypes to look for evidence of excessive apoptosis. We did not find any evidence for elevated apoptosis in *ced-4(EFH1)* using these measures, which suggests that although *ced-4(EFH1)* increased regeneration, this allele does not increase apoptosis.

Mean ± SEM of brood size **(a)** or percentage of dead embryos **(b)** from each animal, n > 8, ns (not significant), * $p < 0.05$, ** $p < 0.01$ or **** $p < 0.0001$ in one-way ANOVA with Tukey adjustment for multiple comparison. **c** is mean ± SEM counts of apoptotic corpses in each L1 animal of indicated genotypes, n > 25, ns (not significant), * $p < 0.05$ or ** $p < 0.01$ in one-way ANOVA with Tukey adjustment for multiple comparison.

Supplementary Figure 5

a

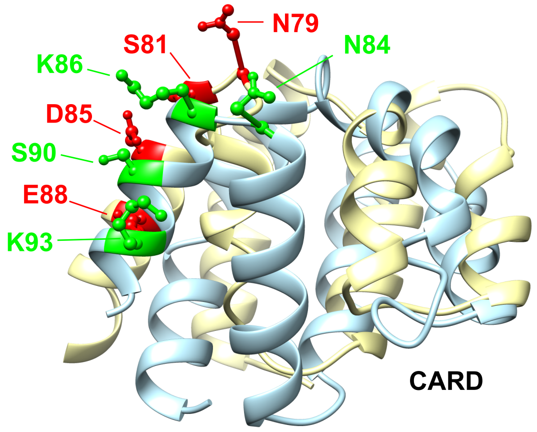

CED-4 (*C. elegans*) vs. DARK (fly)

b

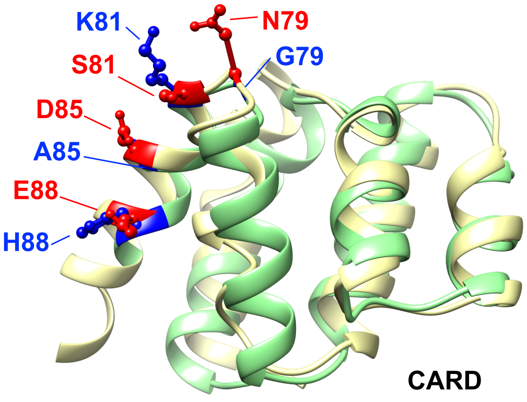

CED-4 (*C. elegans*) vs. Apaf-1 (human)

c

```
C. brenneri (CBN03212)     --------------------------MLHDIERRALEAAHPMLIEDLEPRDALTYLKDQN   34
C. brenneri (CBN29155)     --------------------------MLHDIERRALEAAHPMLIEDFEPRDALTYLKDQN   34
C. brenneri (CBN13342)     --------------------------MLCEIECRALNVAHPMLIQDFEPRDALTYLEALM   34
C. brenneri (Cbn-CED-4)    --------------------------MLCEIECRALNVAHPMLIQDFEPRDALTYLEALM   34
C. japonica (CJA24916)     --------------------------MLVEIECRALNAAHEMLVQDFEPRDALNYLDPLG   34
C. remanei (CRE20313)      ------------------------------------------------------------   0
C. elegans (CED-4)         --------------------------MLCEIECRALSTAHTRLIHDFEPRDALTYLEGKN   34
C. remanei (Cre-CED-4)     MQWFISMQLPFRKYYYDIFSVFSLFQMLCEIECRALNAAHTMLIQDFEPKDALTYLEGQK   60
C. briggsae (Cbr-CED-4)    --------------------------MLCEIECRALNAAHTMLIQDFEPRDALTYLEGEK   34


C. brenneri (CBN03212)     ISTEEHLDLISSMPTRPERIAQFLRAYRKQASALAPLIDFFVYNSQFHFSDFFEERLSIA   94
C. brenneri (CBN29155)     IFTEDHLDLISSMPTRPERIAQFLRAYRKQASALAPLIDFFVYNSQFHLSDFFEERLSIA   94
C. brenneri (CBN13342)     IFSEDHTDQIKAMTTRCGRIAEFLRSYRRQASELAPLIEFFKYNHQTHLSDFFENYIEEA   94
C. brenneri (Cbn-CED-4)    IFSEDHTDQIKAMTTRCGRIAEFLRSYRRQASELAPLIEFFKYNHQTHLSDFFENYIEEA   94
C. japonica (CJA24916)     IFTEDNTDLIINMSTRLERIANFLRGYRRHATELGPLIGFFEQNKQHHIADFLNDYIQLG   94
C. remanei (CRE20313)      ------------------------------------------------------------   0
C. elegans (CED-4)         IFTEDHSELISKMSTRLERIANFLRIYRRQASELGPLIDFFNYNNQSHLADFLEDYIDFA   94
C. remanei (Cre-CED-4)     IFTEDHSDLIGTMPTRLERIANFLRAYRRQASELGPLIDFFTYNNQSHLAHFLEEYIYFA   120
C. briggsae (Cbr-CED-4)    IFTEDHSDLISNMPTRLERIANFLRAYRRQASELAPLIDFFEYNNQNHLKDFLDEYLWFA   94
```

Figure s5. The CED-4 CARD domain is structurally similar to the CARD domain in other CED-4/Apaf-1 family members.

a. Structure alignment of the CARD domains between CED-4 (golden color, *C. elegans*) and DARK (light blue color, fly). The DARK amino acids (green) with equivalent locations as CED-4 S81 and E88 (red) are hydrophilic but carry positive charges rather than negative charges.

b. Structure alignment of the CARD domains between CED-4 (golden color, *C. elegans*) and Apaf-1 (light green color, human). The Apaf-1 amino acids (blue) with equivalent locations as CED-4 S81 and E88 (red) are hydrophilic but carry positive charges.

c. Sequence alignment (via Clustal Omega) of the CED-4 orthologs in different *Caenorhabditis* species. The amino acids with positions corresponding to *C. elegans* CED-4 S81 and E88 (bold and highlighted) are hydrophilic in most cases but not precisely conserved among *Caenorhabditis* species.

Supplementary Tables

Table s1. The sequence of crRNA and ssDNA. The bold letter indicates the newly introduced restriction enzyme cutting sites.

Name	Sequence	Target
EFH1_crRNA	UAGUUGAAAAGUCGAUGAGGUUUUAGAGCUAUGCUGUUUUG-3'	*ced-4* EFH1
EFH1_ssDNA	AGATCTGGCTCATTTATCGCAAAATCGATGTAGTCGGCGAGGAAATC **GGCCAGGTGGGCC**TGATTGTTGTAATTAAAGAAATCAATGAGTGGTC CAAGTTCAGAAGCTTGACGTCGATA-3'	*ced-4* EFH1
EFH2_crRNA	UAUGAUUUUCUAGAAGCUUAGUUUUAGAGCUAUGCUGUUUUG-3'	*ced-4* EFH2
EFH2_ssDNA	TGCTGCTTCTCAAACATGCGAATTCATTGAAGTGACATCA**CTCGAG**AT CGCCGAATGCTACGACTTCTTGGCCGCTTATGGAATGCCGATGCCTG TTGGAGAAAAAGAAGAAG-3'	*ced-4* EFH2

Table s2. List of strains used in this study.

Strain	Genotype	alternate name
ZB4510	zdIs5 [$P_{mec\text{-}4}$GFP + *lin-15(+)*] I	*ced-4(WT)*
ZB4554	zdIs5 [$P_{mec\text{-}4}$GFP + *lin-15(+)*] I; *ced-4(bz401)* III	*ced-4(EFH1)*
ZB4555	zdIs5 [$P_{mec\text{-}4}$GFP + *lin-15(+)*] I; *ced-4(bz404)* III	*ced-4(Δ)*
ZB4556	zdIs5 [$P_{mec\text{-}4}$GFP + *lin-15(+)*] I; *ced-4(bz406)* III	*ced-4(EFH1+2)*
ZB4557	zdIs5 [$P_{mec\text{-}4}$GFP + *lin-15(+)*] I; *ced-4(bz410)* III	*ced-4(EFH2)*
ZB4600	zdIs5 [$P_{mec\text{-}4}$GFP + *lin-15(+)*] I; *ced-4(bz401)* III; *ced-3(n2433)* IV	*ced-4(EFH1); ced-3(lf)*
ZB4019	bzIs178 [$P_{mec\text{-}4}$CA-GFP + $P_{mec\text{-}4}$mCherry]	wild type
ZB4566	bzIs178 [$P_{mec\text{-}4}$CA-GFP + $P_{mec\text{-}4}$mCherry]; *ced-4(bz401)* III	*ced-4(EFH1)*
ZB4021	bzIs178 [$P_{mec\text{-}4}$CA-GFP + $P_{mec\text{-}4}$mCherry]; *ced-3(n2452)* IV	*ced-3(lf)*
ZB4022	bzIs178 [$P_{mec\text{-}4}$CA-GFP + $P_{mec\text{-}4}$mCherry]; *ced-4(n1162)* III	*ced-4(lf)*
ZB4590	zdIs5 [$P_{mec\text{-}4}$GFP + *lin-15(+)*] I; *ced-3(n2433)* IV	*ced-3(lf)*

Supplementary References

1 Yuan, J. & Horvitz, H. R. The Caenorhabditis elegans cell death gene ced-4 encodes a novel protein and is expressed during the period of extensive programmed cell death. *Development* **116**, 309-320 (1992).

2 Li, H., Zhang, L., Rao, A., Harrison, S. C. & Hogan, P. G. Structure of calcineurin in complex with PVIVIT peptide: portrait of a low-affinity signalling interaction. *Journal of molecular biology* **369**, 1296-1306, doi:10.1016/j.jmb.2007.04.032 (2007).

3 Huang, W. *et al.* Mechanistic insights into CED-4-mediated activation of CED-3. *Genes Dev* **27**, 2039-2048, doi:10.1101/gad.224428.113 (2013).

4 Gifford, J. L., Walsh, M. P. & Vogel, H. J. Structures and metal-ion-binding properties of the Ca2+-binding helix-loop-helix EF-hand motifs. *The Biochemical journal* **405**, 199-221, doi:10.1042/BJ20070255 (2007).

5 Hengartner, M. O., Ellis, R. E. & Horvitz, H. R. Caenorhabditis elegans gene ced-9 protects cells from programmed cell death. *Nature* **356**, 494-499, doi:10.1038/356494a0 (1992).
